# High procalcitonin in Gram-negative urosepsis: indicator of immune modulation rather than poor outcome

**DOI:** 10.1007/s00345-026-06199-2

**Published:** 2026-02-27

**Authors:** Sebastian Petzoldt, Christina Buschhaus, Matthias Hecker, Arne Hauptmann, Florian Wagenlehner, Matthias Wolff, Martin Reichert, Veronika Grau, Andreas Hecker, Markus Weigand, Anca-Laura Amati

**Affiliations:** 1 Department of Anesthesiology and Intensive Care Medicine, Giessen University Hospital, Justus-Liebig-University, Rudolf-Buchheim-Strasse 7, 35392 Giessen, Germany; 2 Department of General, Visceral, Thoracic and Transplant Surgery, Giessen University Hospital, Justus-Liebig-University, Giessen, Germany; 3 Department of Internal Medicine II – Pulmonology, Giessen University Hospital, Justus-Liebig-University, Giessen, Germany; 4 Department of Urology, Pediatric Urology and Andrology, Giessen University Hospital, Justus-Liebig-University, Giessen, Germany; 5 Department of Anesthesiology, Medical Faculty Heidelberg, Heidelberg University, Heidelberg, Germany

**Keywords:** Procalcitonin, Gram-negative urosepsis, Interleukin-1β, Immune modulation, Sepsis severity

## Abstract

**Purpose:**

Procalcitonin (PCT) is an acute-phase protein and widely used marker for diagnosing bacterial infection and sepsis, but its physiological role remains incompletely defined. Interleukin-1β (IL-1β) is a critical mediator of the immune response to infection, whose excessive release can drive remote organ injury and dysfunction. Its secretion is therefore tightly controlled. Because other acute-phase proteins have been shown to regulate IL-1β secretion, we investigated whether PCT exerts a similar immunomodulatory effect and whether this influences sepsis severity, particularly in Gram-negative urosepsis.

**Methods:**

Primary human mononuclear leukocytes were stimulated to induce IL-1β release in the presence or absence of increasing PCT concentrations. In parallel, peak PCT levels, infection source, and causative pathogen were analyzed retrospectively in uroseptic patients in comparison to other septic sources, and related to Sepsis-related Organ Failure Assessment (SOFA) scores and serum lactate concentrations.

**Results:**

PCT significantly inhibited IL-1β secretion from primary mononuclear leukocytes across the 2.5–75 µg/L concentration range. Clinically, the highest PCT peaks occurred in patients with Gram-negative urosepsis. Among these, those with peak PCT values within 2.5–75 µg/L had significantly lower SOFA scores and lactate levels—established indicators of sepsis severity and prognosis—compared with patients outside this range.

**Conclusion:**

Within a defined concentration range, PCT down-regulates IL-1β secretion and is associated with reduced markers of disease severity in Gram-negative urosepsis, compared to other sepsis entities. These findings suggest that pronounced PCT elevations in this setting may represent a protective host response rather than a worse prognosis, pointing to a novel immunomodulatory role of PCT in urosepsis that warrants further investigation.

DRKS00037950, retrospectively registered on 22 September 2025.

**Supplementary Information:**

The online version contains supplementary material available at 10.1007/s00345-026-06199-2.

## Introduction

The urinary tract is amongst the most frequent infection sites causing sepsis, with urosepsis constituting the leading cause of sepsis in patients older than 65 years [1]. Procalcitonin (PCT) is widely used as a biomarker for diagnosing and monitoring bacterial infections and sepsis. Unlike C-reactive protein (CRP), serum amyloid A, or interleukin (IL)−6, PCT more effectively distinguishes bacterial from sterile inflammation [2–4]. Elevated PCT levels are strongly associated with Gram-negative pathogens, which cause about 85% of urosepsis cases [5–9]. Accordingly, PCT measurement is recommended by the Surviving Sepsis Campaign to guide the duration of antibiotic therapy [10]. Despite its established clinical utility, the physiological role of PCT in Gram-negative infection remains incompletely understood.

PCT, the prohormone of calcitonin, can increase more than 10,000-fold during bacterial infection [11]. It is produced by nearly all extrathyroidal tissues and white blood cells [7, 12] and appears to exert immunomodulatory effects. Experimental studies indicate both pro- and anti-inflammatory properties. Neutralization of PCT improved survival in sepsis models [13, 14], possibly by counteracting PCT-associated impairment of neutrophil function and endothelial hyperpermeability leading to microcirculatory dysfunction [15, 16]. Conversely, PCT suppresses tumor necrosis factor (TNF) and inducible nitric oxide synthase (iNOS) expression in LPS-stimulated monocytes, implying an anti-inflammatory role [17, 18]. These opposing actions suggest that PCT engages in context-dependent immune regulation shaped by pathogen type, concentration, and host response.

Our group recently showed that acute-phase reactants such as CRP and α₁-antitrypsin inhibit LPS- and ATP-mediated IL-1β release from human monocytes [19–21]. LPS from Gram-negative bacteria and extracellular ATP from damaged cells act as sequential danger signals for inflammasome activation and IL-1β secretion [22, 23], and are both markedly elevated during Gram-negative sepsis [24–26]. Although IL-1β supports host defense, excessive release drives systemic inflammation and multiorgan failure [22, 26, 27]. Given that PCT also behaves as a rapid acute-phase protein [28], we hypothesized that it may similarly modulate IL-1β release. We therefore examined the effect of PCT on LPS and ATP-induced IL-1β secretion from human monocytes in vitro. Further, we analyzed septic patients to determine whether PCT concentrations capable of inhibiting IL-1β correlate with clinical outcomes in Gram-negative urosepsis and compared our findings to abdominal and pulmonary sepsis.

## Methods

This study combined in vitro experiments on human peripheral blood mononuclear cells (PBMCs) with a retrospective analysis of septic patients. Both were approved by the Ethics Committee of the University of Giessen, Germany (Nos. 262/20 and 48/17). The clinical study was registered with the German Clinical Trials Register (DRKS00037950) and followed STROBE guidelines [29].

### Experimental study

PBMCs were isolated from preoperative blood of elective abdominal surgery patients after written consent. Individuals with inflammatory, autoimmune, or chronic infectious diseases or immunosuppression were excluded. PBMCs were separated on Leucosep gradients (Greiner Bio-One, Germany) after priming with 5 ng/ml LPS from *Escherichia coli* (L2654, Sigma-Aldrich, Germany), then stimulated with BzATP (100 µM), an agonist of the ATP-sensitive P2X7 receptor [30], in the presence or absence of recombinant human PCT (R&D Systems, USA). IL-1β levels were measured by ELISA (R&D Systems); cytotoxicity was excluded by lactate dehydrogenase (LDH) assay (Promega, USA).

### Clinical study

This single-center study included adult ICU patients (≥ 18 years) treated for sepsis between 2010 and 2017, fulfilling Sepsis-3 criteria [31]. Patients with confirmed urinary, abdominal, or respiratory infection sites were included; those with unknown or mixed infection sources were excluded. Demographic, clinical, and laboratory data were extracted from prospectively maintained institutional databases, including ICU and hospital length of stay, duration of ventilatory support, need for vasopressor therapy, and ICU mortality. Within the first week of ICU stay, peak lactate levels and maximal SOFA scores—as prognostic indicators of sepsis severity [32, 33]—along with peak PCT and CRP values were recorded. Microbiological data were obtained from site-specific samples: urine cultures for urosepsis, intraabdominal swabs or drain fluid for abdominal sepsis, and tracheobronchial aspirates for pulmonary sepsis. Blood cultures were analyzed in all groups.

### Statistics

Analyses were performed with GraphPad Prism (Version 9 for macOS, GraphPad Software, San Diego, CA, USA, www.graphpad.com). Paired data were analyzed using the Friedman followed by the Wilcoxon signed-rank test. Group comparisons were performed using the Kruskal–Wallis test, followed by the Mann–Whitney rank-sum test when *p* ≤ 0.05. Categorical data used Fisher’s exact or Pearson’s χ² tests. Data are given in mean ± standard deviation (SD) or n (%).

## Results

### Procalcitonin inhibits the BzATP-mediated IL-1β release from human PBMCs

**Fig. 1:**
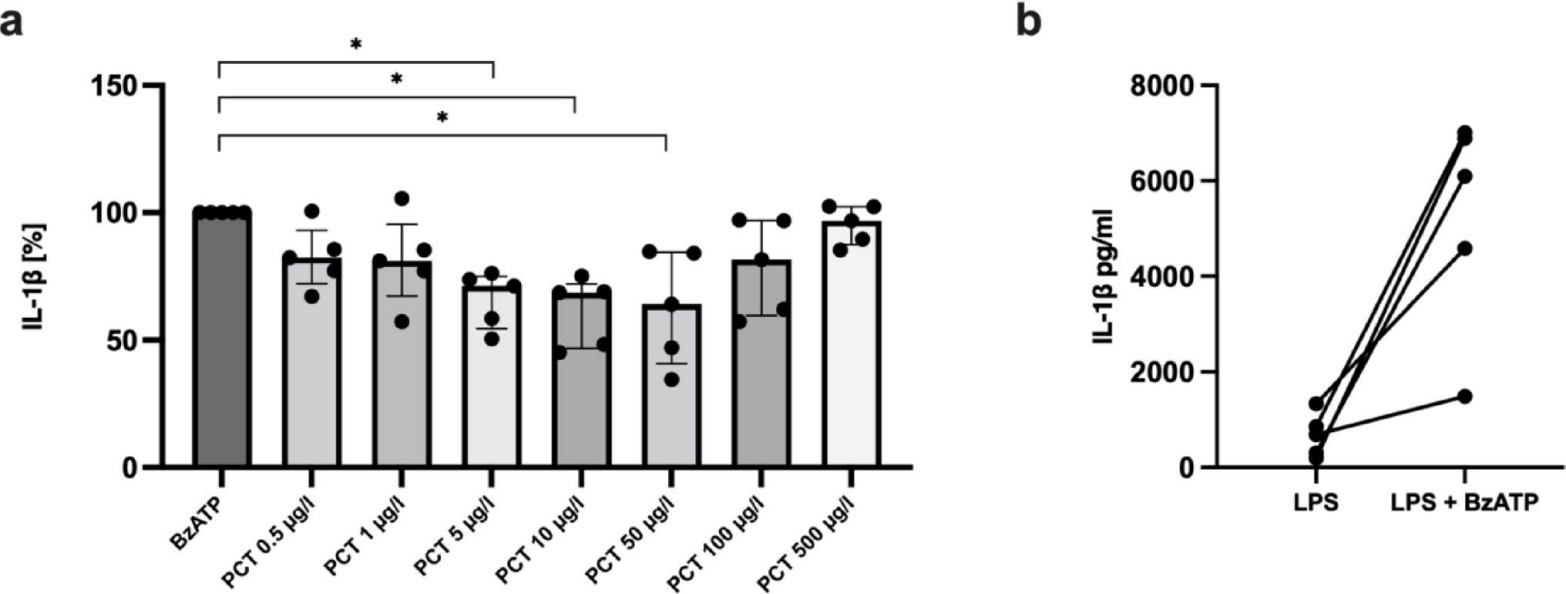
PCT inhibits the LPS and ATP-induced IL-1β release. Freshly isolated peripheral blood mononuclear cells (PBMCs) were primed with lipopolysaccharide (LPS; 5 ng/ml, 3 h) and activated with 2′(3′)-O-(4-benzoylbenzoyl)adenosine-5′-triphosphate (BzATP; 100 µM, 30 min). The release of interleukin (IL)−1β to the cell culture supernatant was measured by enzyme-linked immunosorbent assay (ELISA). **a**. Increasing procalcitonin (PCT) concentrations were added to the LPS-primed cells shortly before stimulation with BzATP. IL-1β levels obtained after stimulation with LPS and BzATP were set to 100%, and all other values were calculated accordingly. Data are presented as individual data points from 5 individual subjects (*n* = 5), the columns represent median, whiskers encompass the 25th to 75th percentile. *p < 0.05, different from cells stimulated with LPS and BzATP, Friedman-test followed by the Wilcoxon signed-rank test. **b**. IL-1β levels after stimulation with LPS alone and with LPS + BzATP, prior to normalization (*n* = 5). Data points from individual patients are connected by lines.

IL-1β levels following stimulation with LPS and BzATP varied between subjects in a range extending from 1490 to 7012 pg/ml (Fig. 1b) and were set to 100%, calculating all other values accordingly. The selected PCT range extended from previously reported diagnostic cut-off values for sepsis (1–2 µg/L) [34] to some of the highest levels observed in septic patients. LDH release remained < 15%, confirming cell viability. At low PCT concentrations (0.5–1 µg/L), no effect was observed, whereas levels between 5 and 50 µg/L reduced LPS- and ATP-induced IL-1β secretion by about 30%; higher concentrations produced no additional inhibition (Fig. 1a).

### PCT in urosepsis compared with abdominal and respiratory sepsis

#### Patient characteristics

Of 683 septic patients screened, 263 met the inclusion criteria, of which 97 had a urinary, 87 an abdominal, and 79 a respiratory infection source (Online Resource 1). BMI and sex distribution were similar, while uroseptic patients were significantly older. Maximal SOFA scores and septic shock incidence did not differ significantly among the sepsis groups. Despite this, urosepsis patients required shorter ICU stays and had the lowest mortality, while pulmonary sepsis patients required longer ventilation and higher vasopressor support, having the highest mortality (Table 1).

**Table 1:** Patient characteristics, microbiologic sampling and outcome. BMI body mass index, SOFA sequential organ failure assessment, ICU intensive care unit. Data are means (± standard deviation) or n (%).^a, b, c^ indicate significant differences between two groups in the Mann–Whitney rank sum test, when the Kruskall–Wallis test for global effects between all three groups was significant

Patient characteristics	Urosepsis (*n* = 78)	Abdominal sepsis (*n* = 87)	Pulmonary sepsis (*n* = 98)	*p*
Demographics				
Male sex, n (%)	47 (60.2%)	48 (55.1%)	68 (69.3%)	0.1293
Age, years (± SD)	71.5 (± 13.7)^a, b^	65.3 (± 15.6)^a^	62.4 (± 15.2)^b^	0.0003
BMI, kg/m^2^ (± SD)	27.4 (± 7.6)	27.0 (± 6.4)	29.3 (± 10.0)	0.5931
Sepsis-related clinical parameters				
Positive microbiologic samples, n (%)	73 (93.5%)	72 (82.7%)	46 (46.9%)	< 0.0001
Positive blood cultures, n (%)	41 (52.5%)	18 (20.6%)	12 (12.2%)	< 0.0001
SOFA, score (± SD)	7.2 (± 3.5)	7.4 (± 3.7)	6.2 (± 2.3)	0.0719
Surgical/interventional procedures, n (%)	43 (55.1%)	86 (98.8)	3 (3.0%)	< 0.0001
Ventilatory support, n (%)	24 (30.7%)	57 (65.5%)	93 (94.8%)	< 0.0001
Maximal noradrenaline perfusion rate, µg/kg/min (± SD)	17.3 (± 23.1)^a, b^	26.4 (± 24,5)^a^	55.5 (± 76.8)^b^	< 0.0001
Septic shock, n (%)	66 (84.6%)	63 (72.4%)	73 (74.4%)	0.1375
Peak PCT range (µg/l)				
IL-1β inhibitory range (2.5–75 µg/l), n (%)	49 (62.8%)	64 (73.6%)	40 (45.5%)	< 0.0001
with confirmed Gram-negative infection, n (%)	36 (46.1%)	35 (40.2%)	8 (8.1%)	
IL-1β non-inhibitory range (< 2.5 or > 75 µg/L), n (%)	29 (37.2%)	23 (26.4%)	48 (54.5%)	< 0.0001
with confirmed Gram-negative infection, n (%)	25 (32%)	6 (6.9%)	12 (12.2%)	
Outcome parameters				
ICU length of stay, days (± SD)	8.2 (± 11.2)^a, b^	17.9 (± 24.3)^a, c^	35.6 (± 43.5)^b, c^	< 0.0001
In-hospital length of stay, days (± SD)	19.10 (± 18.4)^a, b^	41.7 (± 35.6)^a^	40.0 (± 43.8)^b^	< 0.0001
ICU mortality, n (%)	10 (12.8%)	20 (22.9%)	50 (51.0%)	< 0.0001

#### Peak PCT levels in urosepsis compared with abdominal and pulmonary sepsis

**Fig. 2:**
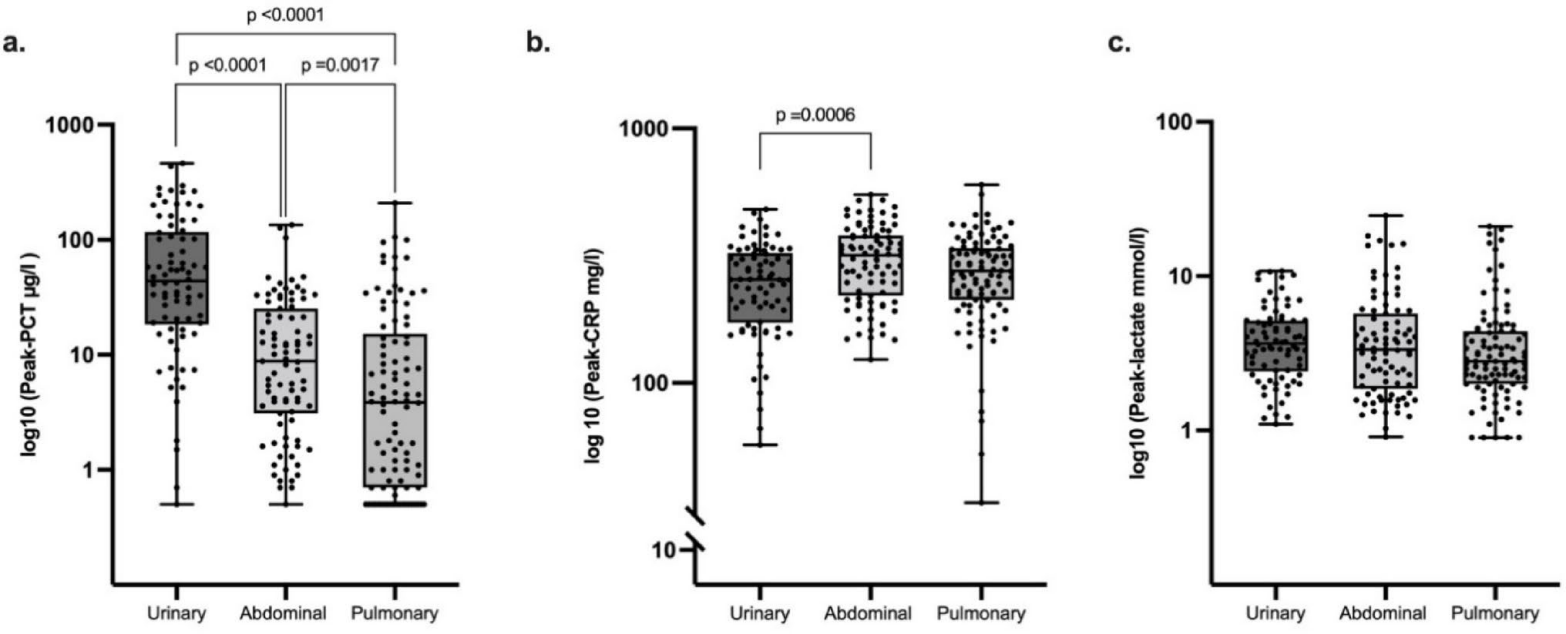
Peak-procalcitonin (PCT), C-reactive protein (CRP) and serum lactate levels in septic patients with urinary, abdominal and pulmonary infection. **a** peak-PCT levels **b** peak-CRP levels and **c** peak-lactate levels. Data are presented as individual data points in logarithmic scale, boxes extend between the 25th and 75th percentile and whiskers between the minimal and the maximal value. The lines in the middle of the boxes are plotted at the median. Significant differences between two groups are indicated when *p* ≤ 0.05 in the Mann–Whitney rank sum test, following the Kruskall–Wallis test with *p* ≤ 0.05

Peak PCT differed significantly among groups and was highest in urosepsis (84.1 µg/L) and lowest in pulmonary sepsis (15.9 µg/L; *p* < 0.0001; Fig. 2a). Peak CRP was highest in abdominal sepsis (312 mg/L) versus urosepsis (253 mg/L; *p* = 0.0006; Fig. 2b), possibly reflecting additional surgical trauma from source-control procedures specific to this group. Peak lactate levels showed no significant differences (Fig. 2c).

#### Microbiological findings in relation to peak PCT values

Gram-negative bacteria predominated in urosepsis—*E. coli* (33.3%), *Proteus* (7.7%), *Pseudomonas* (6.4%)—while abdominal sepsis revealed similar proportions of Gram-negative (24.1%), Gram-positive (29.9%), and mixed infections (26.4%) (Table 2). The highest peak PCT levels occurred in urosepsis with confirmed Gram-negative infection. Only isolated cases of abdominal and pulmonary sepsis exceeded the upper threshold of 75 µg/L (Online Resource 2).

**Table 2:**
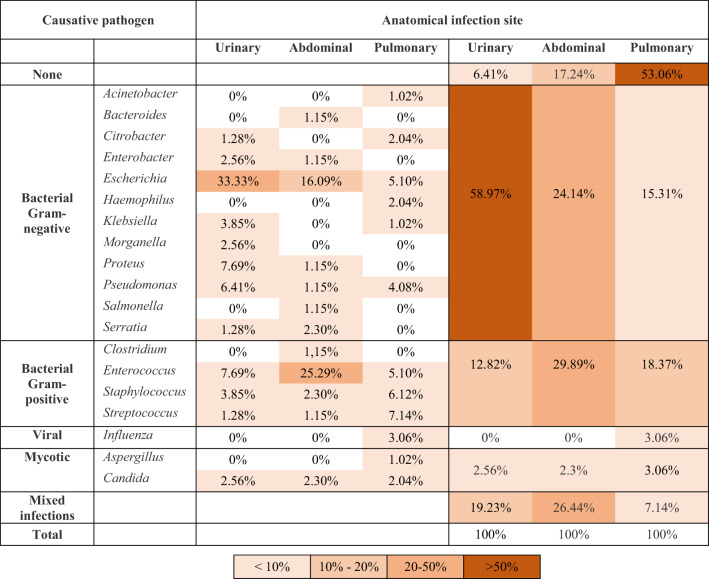
Pathogen spectrum in source-oriented sampling and blood cultures. Percentage from the total number of patients per sepsis group (urinary, abdominal, pulmonary) having a microbiologically confirmed infection with the specified pathogens

#### Reduced severity of Gram-negative urosepsis in patients with PCT in the anti-inflammatory range

**Fig. 3:**
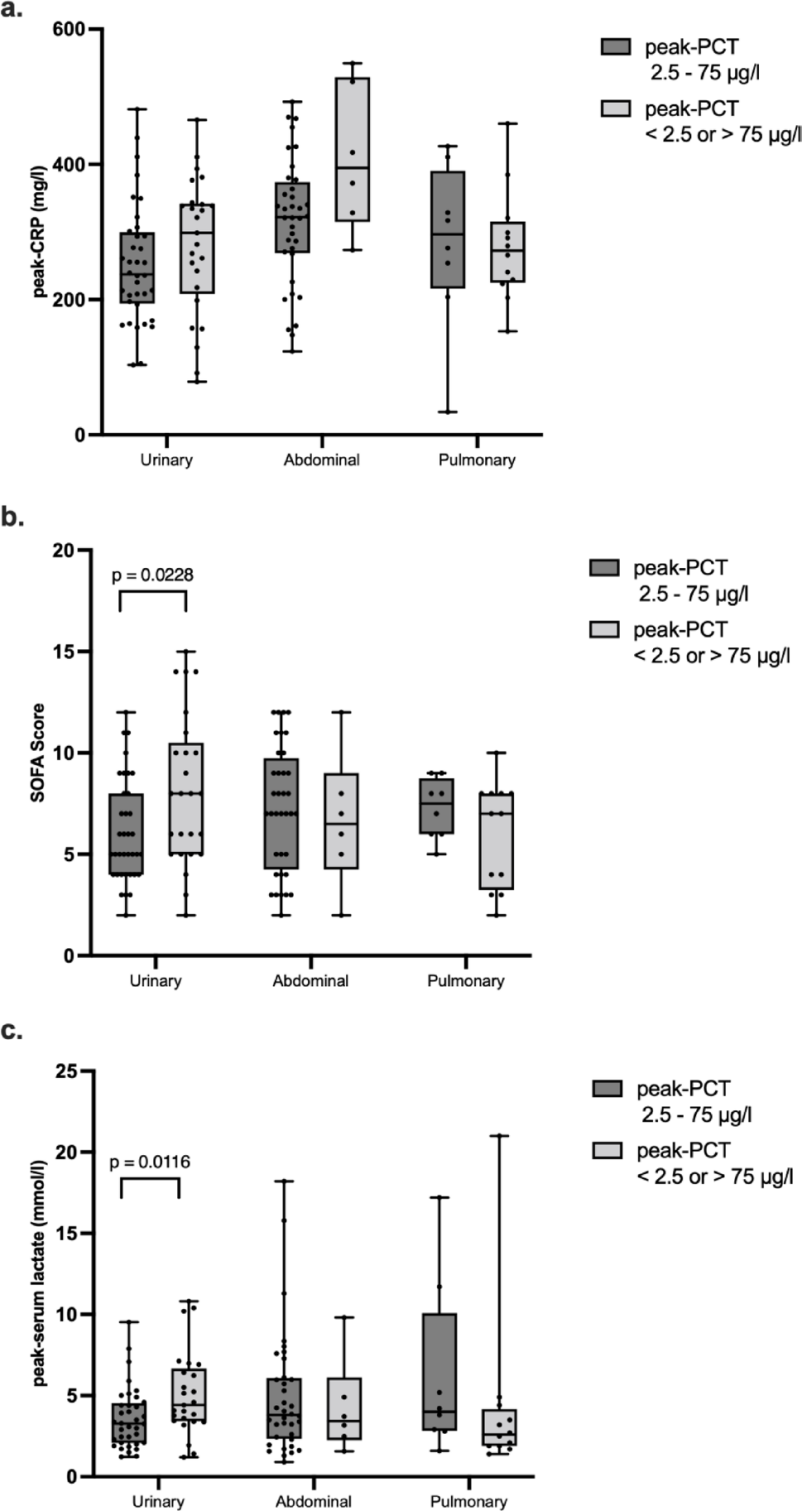
Outcome parameters of patients with Gram-negative sepsis of urinary, abdominal or pulmonary focus with peak-procalcitonin (PCT) values within and outside the IL-1β inhibitory range **a** peak C-reactive protein (CRP) values **b** peak organ failure assessment (SOFA) score **c** peak serum lactate levels. Data are presented as individual data points, boxes extend between the 25th and 75th percentile and whiskers between the minimal and the maximal value. The lines in the middle of the boxes are plotted at the median. Significant differences between two groups are indicated when *p* ≤ 0.05 in the Mann–Whitney rank sum test

Among patients with Gram-negative urosepsis, 36 had peak PCT within the in vitro–observed anti-inflammatory range (2.5–75 µg/L) and 25 outside it. Those within this range showed significantly lower SOFA scores (*p* = 0.0228) and peak lactate levels (*p* = 0.0116)—both established predictors of sepsis severity and outcome [32, 33] (Fig. 3b, c). Notably, in both subgroups, mean peak lactate levels exceeded the 2 mmol/l threshold defined by the Sepsis-3 criteria for septic shock [32]. (Fig. 3b, c). Peak CRP did not differ between sepsis subgroups (Fig. 3a). In Gram-negative abdominal or pulmonary sepsis, SOFA and peak lactate levels were unaffected by PCT range.

## Discussion

This study demonstrates that PCT exerts an immunoregulatory effect, inhibiting IL-1β release from human PBMCs within the 2.5–75 µg/L range frequently observed in urosepsis and other forms of sepsis. Anti-inflammatory effects of PCT have been described previously [17, 35, 36]. For example, PCT at concentrations of 50–500 µg/L decreased LPS-induced TNF and MCP-1 secretion [35], and PCT administration modestly blunted IL-1β in septic animals but not in controls [36].

Our in vitro experiments should be regarded as a pilot study exploring how clinically relevant PCT levels affect IL-1β release. While mechanistic clarification was beyond the scope of this study, the inhibitory effect may share features with other P2X7-related pathways previously described. During inflammation, PCT primarily signals through the calcitonin gene-related peptide receptor [37, 38], a heteromeric G-protein–coupled receptor (GPCR) expressed on monocytes and macrophages [39, 40]. Chemokines such as CCL3–5 and β-nicotinamide adenine dinucleotide have also been shown to inhibit LPS- and ATP-dependent IL-1β release via GPCR-mediated mechanisms [41, 42]. Whether PCT engages a similar signaling route warrants further investigation.

The retrospective analysis examined whether PCT within the IL-1β-inhibitory range correlates with better outcomes. Previous studies have shown that PCT dynamics depend on the infection source and causative microorganism, with higher concentrations observed in abdominal and urinary sepsis, whereas bronchopulmonary infections are associated with a more modest PCT increase [7, 8, 34]. Our results align with these findings, showing that uroseptic patients—despite reaching the highest peak PCT levels—had the lowest mortality and shortest ICU stay. Peak CRP and lactate levels failed to discriminate between infection sources or outcomes. Nearly all cases with very high peak PCT levels and confirmed Gram-negative infection (*E. coli*, *Proteus*, *Pseudomonas*) belonged to the urosepsis group, consistent with data linking PCT elevation to LPS-driven TLR4/NF-κB activation in Gram-negative sepsis [7, 8, 34, 43].

In addition to urosepsis, we included other infectious sources resulting in sepsis for comparison. We demonstrate that urosepsis is dominated by Gram-negative bacteria, which are in turn the main inducers of increased PCT plasma levels. Importantly, within the uroseptic patient group, those with PCT levels between 2.5 and 75 µg/L—matching the inhibitory range identified in vitro—had lower SOFA scores and lactatemia, indicating less severe disease. We provide an approach explaining why the outcome of urosepsis patients is better in comparison to sepsis of other causes. To our knowledge, this is the first study linking moderate PCT elevation with potentially protective immune modulation in Gram-negative urosepsis.

Although PCT’s diagnostic use in detecting urosepsis risk has been extensively studied, its prognostic relevance remains limited [44–47]. Jiang et al. found PCT > 21 µg/L predicted mortality in urosepsis with 73% sensitivity and 83% specificity [47], higher than cutoff values reported in most heterogeneous sepsis cohorts (0.12–14.3 µg/L, pooled sensitivity 76%, specificity 64%) [48]. It is important to note that in most studies, the timing of PCT measurements varied widely, with early levels showing limited prognostic value in sepsis [46]. Measuring peak concentrations during the disease course may provide a more reliable indicator of outcome.

This study has some limitations. The retrospective design of the clinical study is a limitation but ensured a clearly defined infection focus for all included patients. Another limitation stems from applying the IL-1β–inhibitory PCT range, derived from a simplified in vitro model, to a complex clinical setting. This range may differ under the multifaceted immune interactions of sepsis and requires confirmation in future studies. Our study does not provide clear-cut implications for immediate clinical practice in urosepsis. Rather, it adds another piece to the evolving puzzle of how acute-phase proteins may function within the complex immunological context of sepsis. Given the high prevalence of Gram-negative pathogens in urosepsis and the marked elevation of circulating PCT levels, it might be tempting to consider therapeutic strategies aimed at removing PCT from the circulation, for example by plasmapheresis. However, our data suggest—similar to observations reported for other acute-phase reactants [19–21]—that elevated PCT concentrations may constitute part of a negative feedback mechanism contributing to the regulation of inflammatory responses. Consequently, attempts to artificially lower PCT levels in uroseptic patients could be counterproductive or even harmful. Clearly, further experimental and clinical studies are required to validate this concept and to more precisely define the functional role of PCT in urosepsis.

Nonetheless, these findings represent an initial step toward elucidating the molecular mechanisms underlying the pronounced elevation of PCT in urosepseptic patients, underscoring that PCT kinetics and biological effects should be interpreted in relation to the causative pathogen and site of infection rather than extrapolated across unstratified sepsis cohorts.

## Conclusion

Our in vitro findings indicate that LPS- and ATP-induced IL-1β secretion by human PBMCs is inhibited by PCT concentrations between 2.5 and 75 µg/L. The clinical data align with this observation, suggesting that in Gram-negative urosepsis, PCT levels within this range may exert a protective, immunomodulatory effect rather than correlate with adverse outcomes.

## Supplementary Information

Below is the link to the electronic supplementary material.

Supplementary Material 1

## Data Availability

The datasets generated and/or analyzed during the current study are available from the corresponding author upon reasonable request.
