# Supplementary material for: High procalcitonin in Gram-negative urosepsis: indicator of immune modulation rather than poor outcome
Source: World J Urol. 2026 Feb 27;44(1):207. doi: 10.1007/s00345-026-06199-2 (PMC12948892; doi:10.1007/s00345-026-06199-2)
Supplement: Supplementary file 1 — Supplementary Material 1 [file 345_2026_6199_MOESM1_ESM.pdf]

High procalcitonin in gram-negative urosepsis: indicator of immune modulation rather than poor outcome-

- Supplementary Information (SI)-

Submitted to the **World Journal of Urology**

Sebastian Petzoldt[1], Christina Buschhaus[2], Matthias Hecker[3], Arne Hauptmann[4], Florian Wagenlehner[4], Matthias Wolff [1], Martin Reichert[2], Veronika Grau[2], Andreas Hecker[2], Markus Weigand[5], Anca-Laura Amati[2*]

1 Department of Anesthesiology and Intensive Care Medicine, Giessen University Hospital, Justus-Liebig-University, Giessen, Germany.

2 Department of General, Visceral, Thoracic and Transplant Surgery, Giessen University Hospital, Justus-Liebig-University, Giessen, Germany.

3 Department of Internal Medicine II – Pulmonology, Giessen University Hospital, Justus-Liebig-University, Giessen, Germany.

4 Department of Urology, Pediatric Urology and Andrology, Giessen University Hospital, Justus-Liebig-University, Giessen, Germany.

5 Department of Anesthesiology, Heidelberg University, Medical Faculty Heidelberg, Heidelberg, Germany.

For correspondence: Anca-Laura Amati

Phone: +49 641 985 58748 / Fax: +49 641 985 44709

eMail: Anca-Laura.Amati@chiru.med.uni-giessen.de

postal address: Department of General, Visceral, Thoracic and Transplant Surgery, University Hospital of Giessen, Rudolf-Buchheim-Strasse 7, 35392 Giessen, Germany

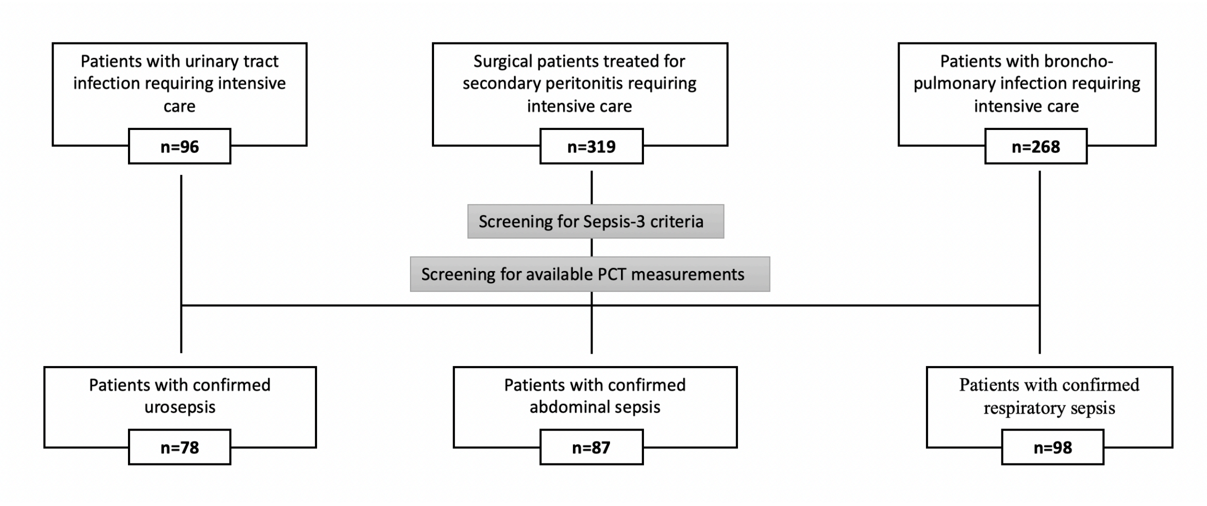

Online Resource 1: Patient selection and subgroup definition. The site of infection for all patients was verified and confirmed by clinical, laboratory, microbiological, imaging or intraoperative findings. All patients with urinary, abdominal or broncho-pulmonary infections that were admitted to one of the intensive care units (ICUs) of our hospital were screened for meeting with the Sepsis-3 criteria and available PCT measurements. All patients under 18 years and patients with an uncertain or other infection sites were excluded from the study

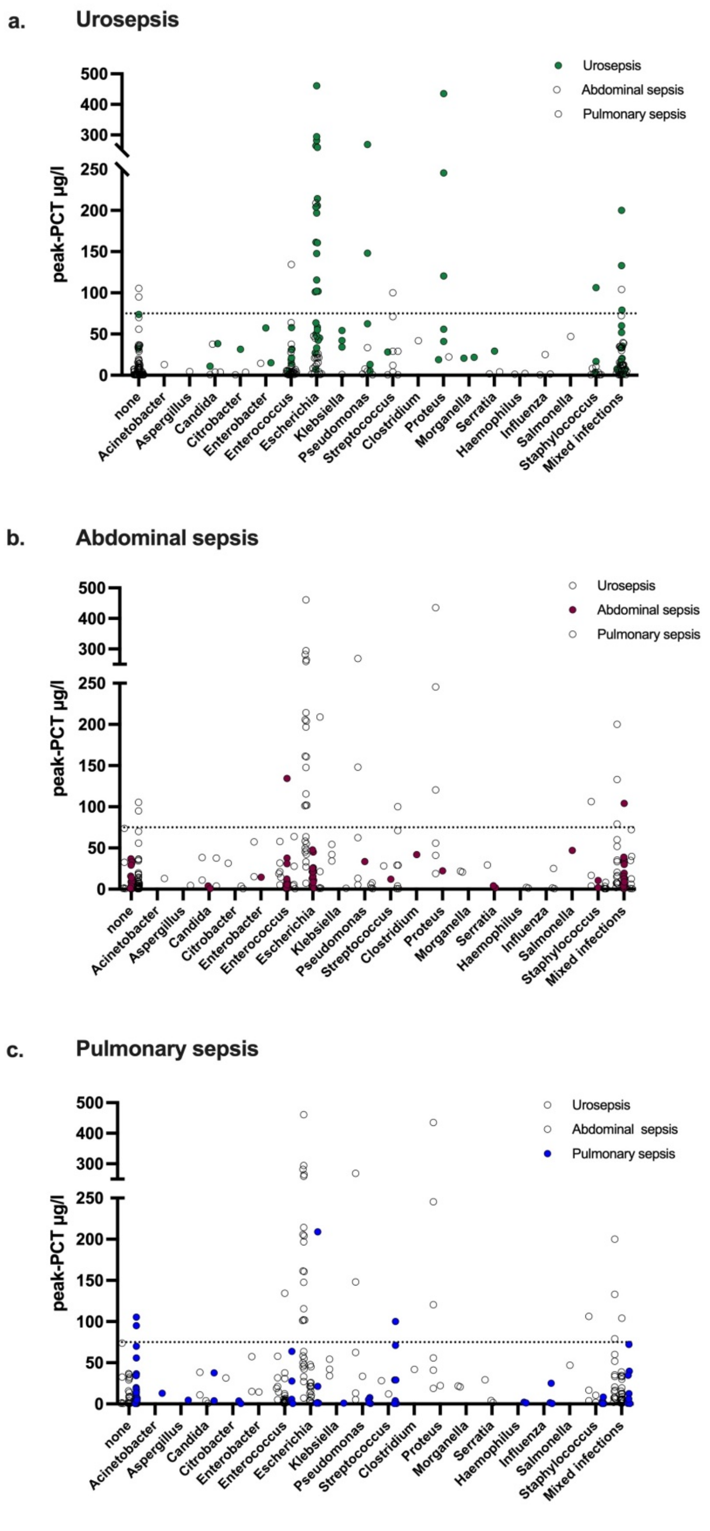

Online Resource 2: **Peak-procalcitonin (PCT) values corresponding to the pathogens identified in source-oriented sampling and blood cultures** in patients with (a.) urosepsis (b.) abdominal sepsis and (c.) pulmonary sepsis.
